# Effect of Inhibiting Butyrylcholinesterase Activity Using Fractionated Coffee Extracts Digested In Vitro in Gastrointestinal Tract: Docking Simulation and Calorimetric and Studies

**DOI:** 10.3390/nu15102366

**Published:** 2023-05-18

**Authors:** Joanna Grzelczyk, Dominik Szwajgier, Ewa Baranowska-Wójcik, Horacio Pérez-Sánchez, Miguel Carmena-Bargueño, Bożena Sosnowska, Grażyna Budryn

**Affiliations:** 1 Institute of Food Technology and Analysis, Faculty of Biotechnology and Food Sciences, Lodz University of Technology, 90-537 Lodz, Poland; grazyna.budryn@p.lodz.pl; 2 Department of Biotechnology, Microbiology and Human Nutrition, University of Life Sciences in Lublin, 20-950 Lublin, Poland; dominik.szwajgier@up.lublin.pl (D.S.); ewa.baranowska@up.lublin.pl (E.B.-W.); bozena.sosnowska@up.lublin.pl (B.S.); 3 Structural Bioinformatics and High-Performance Computing Research Group (BIO-HPC), Computer Engineering Department, Universidad Católica de Murcia (UCAM), Guadalupe, 30107 Murcia, Spain; hperez@ucam.edu (H.P.-S.); mcarmena@alu.ucam.edu (M.C.-B.)

**Keywords:** polyphenol, coffee, chlorogenic acids, in vitro digestion, bioavailability, probiotics, ITC, neurological, chronic disease

## Abstract

Butyrylcholinesterase (BChE) is a major enzyme from the alpha-glycoprotein family that catalyzes the hydrolysis of neurotransmitter acetylcholine (ACh), lowering the concentration of ACh in the nervous system, which could cause aggravation of Alzheimer’s disease (AD). In select pathological conditions, it is beneficial to reduce the activity of this enzyme. The aim of this study was to evaluate the degree of BChE inhibition by coffee extracts fractionated into mono- and diesters of caffeic acid/caffeine, digested in vitro in the gastrointestinal tract. The bioactive compounds from coffee showed high affinity for BchE, −30.23–−15.28 kJ/mol, and was the highest for the caffeine fraction from the green Arabica extract. The isolated fractions were highly effective in inhibiting BChE activity at all in vitro digestion phases. It has been shown that the fractionation of coffee extracts could be potentially used to obtain high prophylactic or even therapeutic effectiveness against AD.

## 1. Introduction

Butyrylcholinesterase (BChE) is a multifunctional enzyme from the alpha-glycoprotein family, produced mainly in the liver, both in hepatocytes and cholangiocytes [1]. Recently, Severi, Abbatelli, Perugini, Di Mercurio, Senzacqua, and Giordano [2] confirmed the presence of BChE-secreting cells in a mouse model in the keratinized layers of the squamous epithelium of the esophagus and forestomach, in the oxyntic mucosa of the stomach, in the mucus-secreting cells of duodenal Brunner glands, and in the small and large intestinal mucosa. This sheds new light on the activity of the enzyme and its role in the regulation of physiological processes. BChE occurs at high concentrations in the liver and nervous system, but its presence in the gastrointestinal tract is not without significance [2,3]. The enzyme promotes lipid metabolism [4]; catalyzes the hydrolysis of various substances including xenobiotics, such as aspirin [1,4], in particular the neurotransmitter acetylcholine (ACh) [3]; and is involved in the hydrolysis of some hormones secreted in the digestive system, responsible for the regulation of carbohydrate–lipid metabolism. In contrast, a mutation of the BChE gene positively correlates with the occurrence of, among others, colorectal cancer [1]. The most understood function of BChE is supporting the degradation of ACh, which, under the influence of hydrolysis, decomposes into acetic acid and choline [4,5]. It occurs when the level of acetylcholinesterase (AChE) is insufficient, which leads to the gradual takeover of ACh degradation by BChE [3]. However, in certain pathological conditions, ACh concentrations can be very low, and high BChE activity may further decrease them. Lowering the level of ACh in the nervous system disrupts brain function and contributes to aggravation of Alzheimer’s disease (AD) [6]. AD patients are mainly treated with inhibitors acting on BChE and AChE or on the AChE enzyme itself. Therefore, an oversupply of the BChE enzyme at a later stage of AD disease is not effectively blocked [7]. Natural compounds that are nontoxic inhibitors of BChE are still being sought [6], and their positive effects can go far beyond the relief of symptoms of neurodegenerative diseases [8]. In recent years, epidemiological studies have shown that regular coffee consumption has been linked to slower cognitive decline, and in in vivo models, the influence of coffee beverages on appetite and satiety regulation, as well as on brain–gut axis functioning, was demonstrated [9,10,11,12,13]. It has been hypothesized that the observed effects are in part linked to the activity of coffee nutraceutics as BChE inhibitors. Their concentration and accessibility may change during digestion depending on many factors. Therefore, this work focuses on the characterization, at the molecular level, of the effect of select bioactive ingredients of coffee extracts on the inhibition of BChE activity after simulated in vitro digestion, which has not yet been completely elucidated.

The aim of this study was to determine how in vitro digestion changes the concentration of bioactive substances in fractionated coffee extracts in various sections of the gastrointestinal tract and how the presence of probiotic bacteria could contribute to these changes. The affinity of coffee components for binding to BChE and the stability of enzyme–ligand (coffee nutraceutics) complexes, resulting in enzyme inhibition, were also investigated based on thermodynamic analyses and molecular simulations, depending on the coffee roasting level and the digestion phase. Since earlier studies by Grzelczyk, Szwajgier, Baranowska-Wójcik, Budryn, Zakłos-Szyda, and Sosnowska [14] showed the described beneficial effects of coffee extracts, we suppose that preparations isolated from coffee extracts may work more intensively and be more useful for the development of functional foods.

## 2. Materials and Methods

### 2.1. Materials and Reagents

Formic acid, acetonitrile, methanol, UHPLC-grade water, porcine pancreas pancreatin, porcine bile extract, ethylenediaminetetraacetic acid, 2-(N-morpholino acid), ethanesulfonic acid, mucin, tris-(hydroxy-methyl) aminomethane, 3,5-dinitrosalicylic acid, trinitrobenzenesulfonic acid, glucose, L-leucine, α-amylase, pepsin, acrylamide (AK, ≥99%), hydroxymethylfurfural (HMF, ≥99%), caffeine (≥99%), caffeic acid (CA, ≥99%), ferulic acid (FA, ≥99%), 3-caffeoylquinic acid (3-CQA), 4-caffeoylquinic acid (4-CQA), 5-caffeoylquinic acid (5-CQA, ≥99%), 3,4-dicaffoylquinic acid (3,4-DCQA, ≥98%), 3,5-dicaffoylquinic acid (3,5-DCQA), 4,5-dicaffoylquinic acid (4,5-DCQA), butyrylcholinesterase (EC 3.1.1.8 from serum horse, freeze-dried powder, ≥10 units/mg protein, BChE), nutrient agar, TOS agar, and MRS agar were purchased from Sigma Aldrich, Merck (St. Louis, MO, USA). Gibson’s solution was prepared according to Szwajgier et al. [15]. Nylon syringe filters were supplied by Chromacol (Herts, UK) [15,16].

The probiotic preparation Jarro-Dophilus EPS, consisting of 9 strains, was purchased from MZ Store (Supps4you LLC, Lewes, DE, USA). One capsule contained a mixture of probiotic bacteria strains: *Lactobacillus rhamnosus* (R0011), *Lactobacillus helveticus* (*L. acidophilus*, R0052), *Bifidobacterium breve* (R0070), *Pediococcus acidilactici* (R1001), *Lactobacillus casei* (R0215), *Lactobacillus plantarum* (R1012), *Lactobacillus lactis* ssp., *lactiss* (R1058), *Pediococcus acidilactici* (R1001), and *Bifidobacterium longum* (BB536).

Arabica and Robusta coffee beans were purchased from Bero Polska (Gdynia, Poland). The beans were roasted to obtain light and dark degrees of roasting. The preparation of coffee extracts and the isolation of fractions were described in previous work by [16]. In short, green beans of Robusta and Arabica coffees were ground and aqueous extracts were obtained via pressure boiling at 110 °C. The extracts were lyophilized. Next, freeze-dried extracts of green, light-, and dark-roasted coffee were fractionated by performing CPC using the Spot Prep II system (Armen, France). Three fractions were collected from the green and roasted coffees: the caffeine fraction, where the alkaloid constituted from 10.01 to 21.90 g/100 g d.b.; the monochlorogenic acids, which contained caffeoyl- and feruloylquinic acids isolated from green and roasted coffees in the range of 8.91–50.41 g/100 g d.b. of the fraction; and the dichlorogenic acids, containing dicaffeoylquinic acids in the range of 3.09–22.33 g/100 g d.b. [16]. The remaining parts were other coffee compounds.

### 2.2. In Vitro Digestion of Fractionated Coffee Extracts

Digestion was carried out according to the method of Grzelczyk et al. [14] with minor modifications. The lyophilized fractionated extracts of green, light-, or dark-roasted coffee in amounts of 1 g were mixed with 200 mL of distilled water. The pH was then adjusted to 1.7 (5 mol/L, HCl), and 200 mg of pepsin was added. Digestion in the mouth was omitted since coffee infusions are not chewed but swallowed immediately after being taken into the mouth. In the next step, the sample was incubated at 37 °C for 1.5 h with continuous stirring (20 rpm). A 0.1 mol/L solution of NaHCO_3_ was then added to correct the pH to 7.5 and to stop the process of lysis by pepsin. Samples were taken at that step to represent the “gastric” phase. Subsequently, 150 mL of Gibson’s solution was added to the sample [15], and the pH was corrected to 6.5 by carefully adding 1 mol/L of a HCl solution. At the same time, 50 mg of pancreatin and 25 mg of bile salt suspended in 1 mL of Gibson’s solution were added. The sample was incubated for 3 h, and the pH was continuously corrected to 6.5 with sterile NaOH or HCl solutions (3%, *w*/*v*) using peristaltic pumps, after which samples were taken to represent the “small intestine” phase. Incubation was then carried out for another 3 h, and samples were taken for analysis to represent the “large intestine” phase. Then, the Jarro-Dophilus EPS vaccine was added, with each of the 9 strains added at a dose of 0.625 × 109 cfu per “digestion”. For this purpose, 10 mL of sterilized and cooled saline (0.9% aqueous NaCl solution) was aseptically added to the bacterial inoculum, thoroughly mixed, and injected into the simulated digestive tract. The system was left overnight, and samples were taken after 9 h to represent the “colon” phase. After collection, each sample was directly heated in a water bath at 65 °C for 15 min to inactivate the enzymes and directly frozen at −80 °C for analysis. Simultaneously, reagent (blank) samples without coffee were run in the same manner. Differences in the volumes of individual “digestions” resulting from different volumes of the used correction solutions (HCl and NaHCO_3_) were bridged by adding distilled sterile water until all volumes were equal. During the whole “digestion” process, sterile CO_2_ (from the cylinder) was passed through the solutions to obtain anaerobic conditions.

### 2.3. Analysis of Chlorogenic Acids and Caffeine by LC-ESI-MS

The profile of chlorogenic acids in fractions from green and roasted coffee extracts released during digestion in a model gastrointestinal tract was analyzed using the UHPLC-ESI-MS technique according to Grzelczyk et al. [14]. Digested samples were injected after proper dilutions depending on the volume of the reagents added in the “digestive tract”. This was conducted to standardize the volume of samples taken at different stages of in vitro digestion, which were characterized by a different degree of dilution of bioactive compounds from coffee, resulting from the continuous addition of new reagents to the system. After standardization, the samples were filtered through a 0.2 µm nylon syringe filter. A chromatographic analysis was performed using a CBM-20A controller, two LC-2020AD pumps, an SIL-30AC autosampler, a CTO-20AC column thermostat (Shimadzu, Tokyo, Japan), a photodiode array detector (SPD-M20A radiant diode array detector, Shimadzu, Tokyo, Japan), and a mass spectrometer (LCMS-2020, Shimadzu, Tokyo, Japan) equipped with an electrospray ionization (ESI) source. Chromatographic separation was performed using a Kinetex EVO C18 Corre-Shell (150 mm × 3.0 mm × 2.6 μm) analytical column from Phenomenex (Torrance, CA, USA), an acetonitrile/ formic acid (99.9:0.1, *v*/*v*) solvent system, and a flow rate of 0.2 mL/min. The injection volume was 2 µL. Identification of chlorogenic acids and caffeine was based on the comparison of the UV and mz spectrum with the appropriate standard [14].

### 2.4. Viability of Bacteria in the In Vitro Digestive System

The viability of bacteria in the simulated digestion system under the influence of coffee nutraceutics was checked according to the procedure described by Grzelczyk et al. [14] with minor modifications. For this purpose, suspension of bacterial cultures prepared directly from the initial Jarrow formula vaccine and from the samples of cultures taken during in vitro digestion after the “large intestine” and “colon” phases were investigated (3 and 12 h) after contact of the probiotic preparations with the coffee fractions or with the reagents in the case of the control sample. Next, microbiological analyses of bacteria of the genera *Lactobacillus* (MRS medium, incubation 48 h, temp. 37 °C, anaerobic conditions), *Lactococcus and Pedicoccus* (medium M17, incubation 48 h, temp. 30 °C, aerobic conditions), and *Bifidobacterium* (Garche’s medium, incubation 48 h, temp. 37 °C, anaerobic conditions) were carried out. Cultures were grown from a series of decimal dilutions and added into two plates flooded with a suitable medium. After incubation, characteristic colonies of individual types of bacteria were counted on each substrate and calculated to give the number of colony-forming units. The results were converted to log10. In order to avoid error, due to the fact that the amount of fluid in the artificial gastrointestinal tract was not always the same, the obtained results were calculated each time based on its proportion to the total volume in which the control sample was digested [14].

### 2.5. Inhibition of BChE Activity Evaluated by Isothermal Titration Calorimetry

The analysis was carried out according to the procedure by Budryn, Grzelczyk, Jaśkiewicz, Żyżelewicz, Pérez-Sánchez, and Cerón-Carrasco [17]. For the analysis of the interactions of the coffee nutraceutics with BChE (EC 3.1.1.8) and the stability of the formed complexes, concentrations of 2 × 10^−6^ mol/L of the enzyme and 2 × 10^−4^ mol/L of the fractions from the coffee extracts (calculated as 5-CQA) from a suitable phase of in vitro digestion with or without bacteria were used, and in a separated test, ACh at a concentration of 3 × 10^−4^ mol/L was added to the syringe and injected along with the coffee nutraceutics to evaluate the inhibition of its hydrolysis. A total number of 11 injections, each of 2 μL volume, was performed in one run within 30 min [17]. During the experiments, changes in enthalpy (∆H); Gibbs free energy, also described as affinity (∆G); entropy (∆S); dissociation constant (KD); and the complexation (association) constant (KA) were determined on the basis of the Gibbs equation. The enzyme inhibition constant (Ki) of ACh hydrolysis catalyzed by BChE in the presence of inhibitors was calculated using the Michaelis–Menten equation and the enzyme reaction model. The data were calculated using MicroCal PEAQ-ITC Analysis software MicroCal PEAQ-ITC200. The “one binding site” interaction model was used. To calculate enzyme activity inhibition, the ACh hydrolysis signals in the presence of inhibitors were subtracted from the ACh hydrolysis signals without inhibitors, taking into account the enthalpy recorded during the interaction of the inhibitors with BChE.

The inhibitory activity was calculated as [(∆H − ∆Hi)/∆H] × 100%, where ∆Hi is the signal of ACh hydrolysis in the presence of the inhibitor (minus the signal of interaction of the inhibitor with the enzyme) and ∆H is the signal of ACh hydrolysis without the inhibitor. The percent inhibition of ACh hydrolysis at a given ligand concentration was calculated and used to calculate the ligand concentration that inhibited the hydrolysis reaction by 50% (IC50) [17].

### 2.6. Molecular Modelling

The characteristic X-ray crystal structure of BChE, coded as 1POI from the Protein Data Bank (PDB) database (http://www.rcsb.org/pdb (accessed on 11 March 2023), was selected for analysis. In the next step, using the Protein Preparation Wizard tool, which is available in the Maestro software version 2020-4 [18], an appropriate enzyme model was prepared; the hierarchy of bonds was established, and hydrogen atoms were added. In this tool, the following options were selected: “Assign bond orders”, “Use CCD database”, “Add hydrogens”, “Create zero-order bonds to metals”, “Create disulfide bonds”, and “Generate heat states using Epik pH 7 ± 2.0” [19]. After that, using the tool System Builder, the charges were assigned based on ForceField OPLS3e [20]. The chemical structures of the chlorogenic acids were built and fully optimized using the tool of Maestro Lig-Prep. This tool uses Epik [19] to calculate the protonation state of each molecule (the pH used was 7 ± 0.5), and it assigns the charges of each atom using ForceField OPLS3e [20]. Docking of the chlorogenic acids to the prepared enzyme model was performed with the Lead Finder docking program [21] using default parameters. The size of the ligand-docking mesh was set as 30 Å in each direction from the geometric center for each individual docking simulation. The evaluation function of the Lead Finder program takes into account the Lennard–Jones factor, hydrogen bonds, electrostatic interactions, stabilizing hydrophobic interactions, and correction of entropy for the number of binding turns and the ligand internal energy. The representation of each interaction was performed using Poseview software version 1.1.2 [22]. All these calculations were run using metascreener (https://github.com/bio-hpc/metascreener, accessed on 1 February 2023). In the current experiment, it was verified whether the hierarchy of the attachment of individual nutraceuticals from the given fraction to the enzyme could influence the degree of its inhibition and whether the compounds are competitive in binding the catalytic site of BChE.

### 2.7. Statistical Analysis

The statistical analysis consisted of determining the mean values of six measurements and their standard deviation (±SD) and of a unidirectional analysis of variability, using Statistica 10.0 from StatSoft (Inc., Tulsa, OK, USA) at a significance level of *p* < 0.05. Significant differences between mean values were estimated using the Tukey statistically significant difference test. In the microbiological analysis, 4 repetitions were performed.

## 3. Results and Discussion

### 3.1. The Effect of Different Fractions from Coffee Extracts on the Viability of Probiotic Lactic Acid Bacteria during In Vitro Digestion

**Figure 1:**
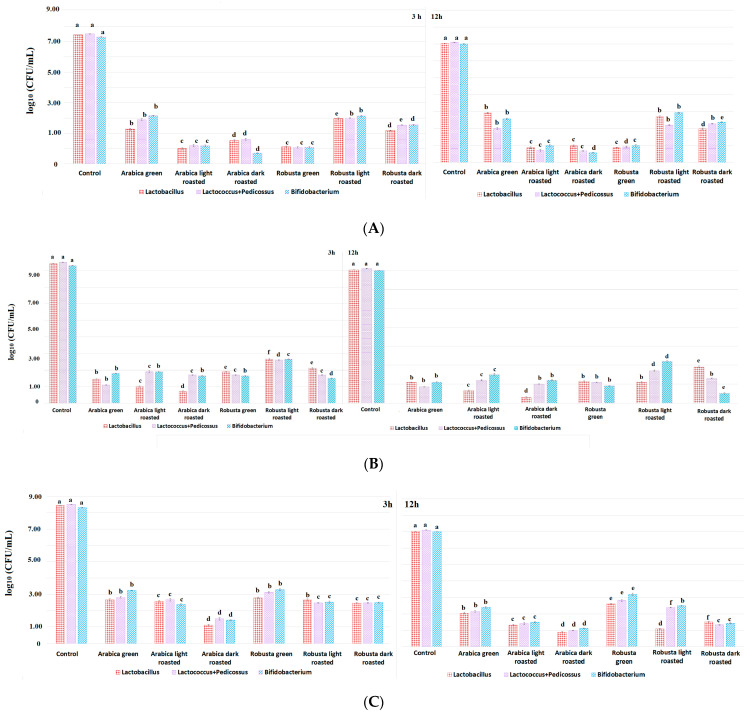
Viability of lactic acid bacteria during in vitro digestion. Mean values of log10 (CFU/mL) obtained for the control and fractions of coffee extract, in vitro large intestine digestion after 3 h, and in vitro colon digestion after 12 h. (**A**) Caffeine fractions; (**B**) fraction of monochlorogenic acids; (**C**) fraction of dichlorogenic acids. ^a–f^ The significant difference (*p* < 0.05) of the different bacteria with coffee compared with control without coffee. For each of the bacteria, the statistics for the control sample without coffee fractions and with coffee fractions with different degrees of roasting were analyzed separately.

The viability of tested strains of probiotic lactic acid bacteria (LAB) during in vitro intestinal digestion was investigated in the presence of fractions from coffee extracts—mono- and dichlorogenic acids, as well as caffeine—and in the control protocol, only under the influence of substances used to perform the digestion simulation. The results of the analyses are presented in Figure 1A–C. All analyzed fractions of coffee extracts resulted in a reduction in the survival of LAB during in vitro digestion. The results suggest that the highest survival level of LAB occurred on average during digestion with the dichlorogenic acid fraction, followed by caffeine, while the highest antimicrobial activity was shown for the monochlorogenic acid fractions.

Bacterial survival in the presence of caffeine fractions isolated from coffee extracts showed a similar trend regardless the stage of digestion (Figure 1A). The most beneficial properties affecting the growth of bacteria after 3 and 12 h of digestion were characterized by the fraction from green Arabica, as well as light- and dark-roasted Robusta, maintaining a bacterial count at a level of about 2–3 log10 (cfu/mL). On the other hand, the worst survival characteristics occurred in the presence of dark-roasted Arabica, where the number of LAB was lowered to 0.6–1.7 log10 (cfu/mL).

Bacterial survival in the presence of the isolated fraction of monochlorogenic acids decreased with the digestion duration (Figure 1B). In samples taken after the large intestine phase, survival was the highest in the presence of the fraction from light-roasted Robusta, resulting in an amount of 2.7 log10 (cfu/mL). Monochlorogenic acids from the other stages of roasting of Robusta and Arabica contributed to survival determined at the level of 0.7–2.1 log10 (cfu/mL). However, after 12 h of digestion of the monochlorogenic acid fractions, the highest survival rate was assigned for LAB in the presence of light- and dark-roasted Robusta (1.0–2.2 log10 cfu/mL), while the lowest survival rate was associated with the presence of dark-roasted Arabica (0.3–1.2 log10 cfu/mL). The highest viability during the digestion concerned *Bifidobacterium*, in the cases of both caffeine and monochlorogenic acid fractions of coffee extracts.

The highest survival rate was observed in the presence of dichlorogenic acid fractions, suggesting better prebiotic properties in these compounds (Figure 1C) compared with the caffeine and monochlorogenic acid fractions. It was observed that dichlorogenic acids from both green Robusta and, to a lesser extent, green Arabica showed the best prebiotic effect. After 3 h of digestion for *Bifidobacterium*, the viability was 3.2 log10 (cfu/mL) in the presence of green Robusta and Arabica, while for *Lactococcus + Pediococcus*, it was 3.1 and 2.8 log10 (cfu/mL), respectively, and, for *Lactobacillus*, it was 2.8 and 2.7 log10 (cfu/mL). In the samples taken after 12 h, it can be seen that the survival rate of bacteria was still at a high level compared with the result obtained after 3 h of digestion. Taking into account the roasting level, the weakest antimicrobial properties were found with the fraction of green and light-roasted Robusta, and the strongest properties were found with the dark-roasted Arabica. Mills et al. [23] suggested that 5-O-caffeoylquinic acid (5-CQA) selectively modulates the microbiota in the colon in vivo. This is due to the fact that about one-third of CQA is absorbed in the small intestine only, while two-thirds of this compound enter the large intestine unchanged, where they are metabolized by colonic bacteria. 5-CQA can come from the gradual hydrolysis of dichlorogenic acids, explaining the favorable properties of diesters. The authors suggest a significant positive effect of CQA on the growth of *Bifidobacterium* spp. In other studies, the administration of 5-CQA positively affected the growth of *Bifidobacterium* spp., while it did not affect the growth of *Lactobacillus* [24]. Parkar, Trower, and Stevenson [25] also showed that gut microbes such as Lactobacillus and Bifidobacterium can be involved in the metabolism of CQA to quinic acid and CA, which is further metabolized to 3-hydroxycinnamic, 3,4-dihydroxyphenylacetic, and 3-hydroxyphenylacetic acids and then absorbed in the intestine by monocarboxylic transporters. Sales et al. [26] evaluated the effect of coffee species, roasting degree, and caffeine content on the growth of probiotic bacteria in vitro. They have shown that the addition of 0.5–1.5 g/100 mL of extracts from roasted Arabica and Robusta or 0.05–0.8 mg/mL of caffeine, trigonelline, arabinogalactan, and galactomannan resulted in an increase in the count of *Bifidobacterium* spp. and *Lactobacillus rhamnosus* cells by log10, resulting in an amount of 0.5–1.8 cfu/mL in the case of medium-roasted beans, while dark-roasted coffee caused an increase of 0.9–1.7 cfu/mL. Interestingly, caffeine limited the growth of *Lactobacillus rhamnosus* (R0011). It must be noted that our study was conducted at low prebiotic contributions in the fractions, while coffee extracts contain a significant amount of dietary fiber that acts as a prebiotic and can alleviate the antimicrobial properties of coffee phenolic acids or caffeine. A similar effect could possibly be observed in the case of functional foods rich in fiber and supplemented with health-promoting coffee fractions. Therefore, the effect of reducing the number of probiotics using caffeine or chlorogenic acids in the intestine in the presence of prebiotics may ultimately be insignificant [27].

### 3.2. Profile of Chlorogenic Acids and Caffeine in Fractions from Coffee Extracts after In Vitro Digestion

**Figure 2:**
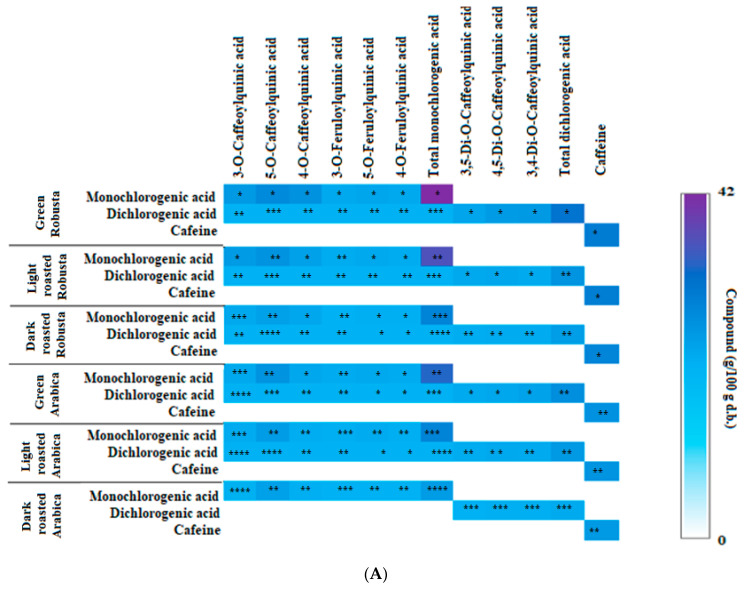

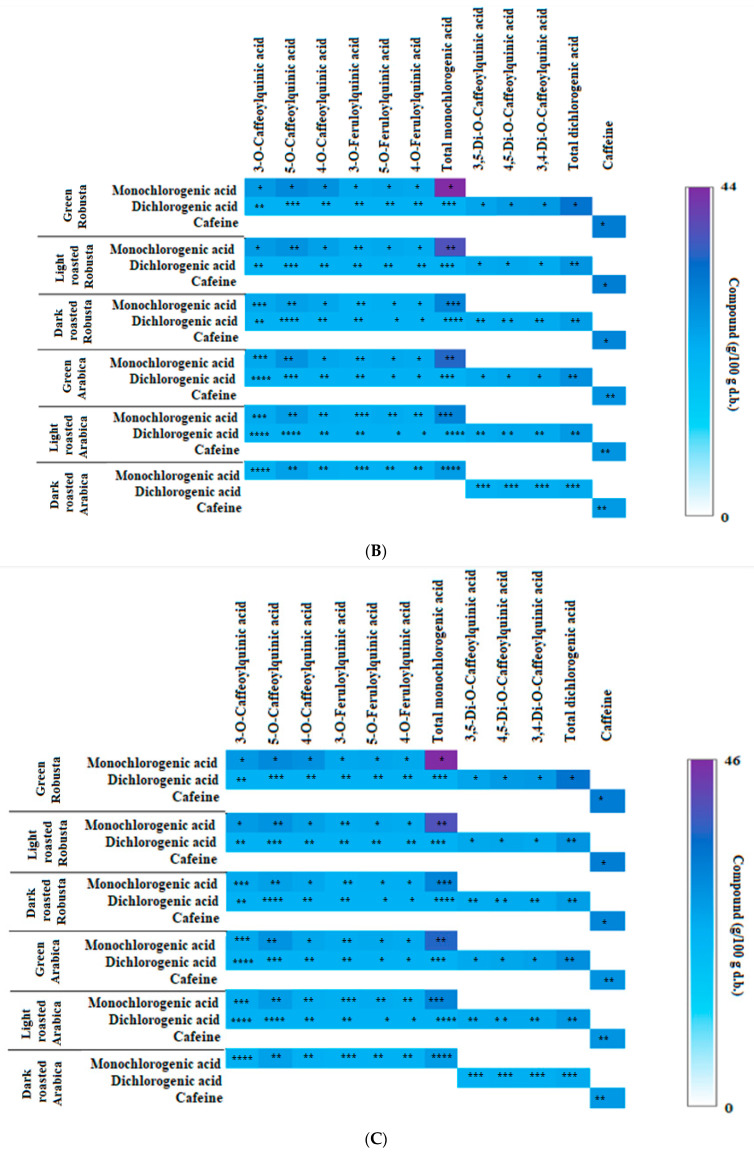

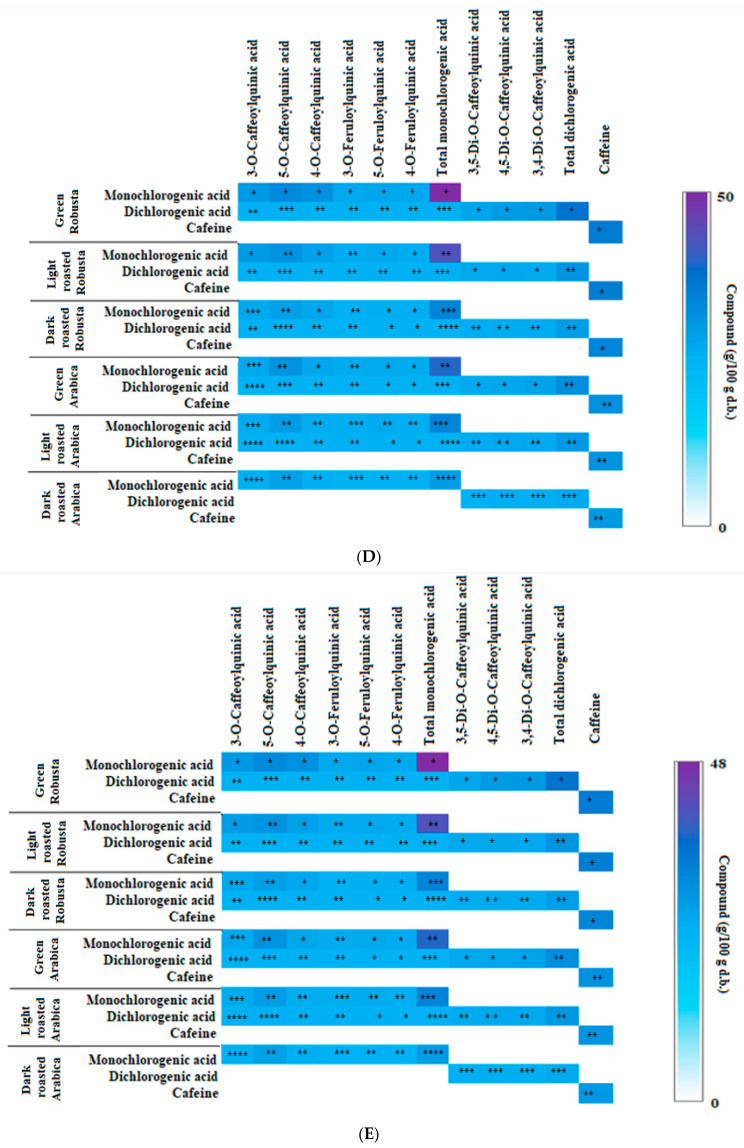

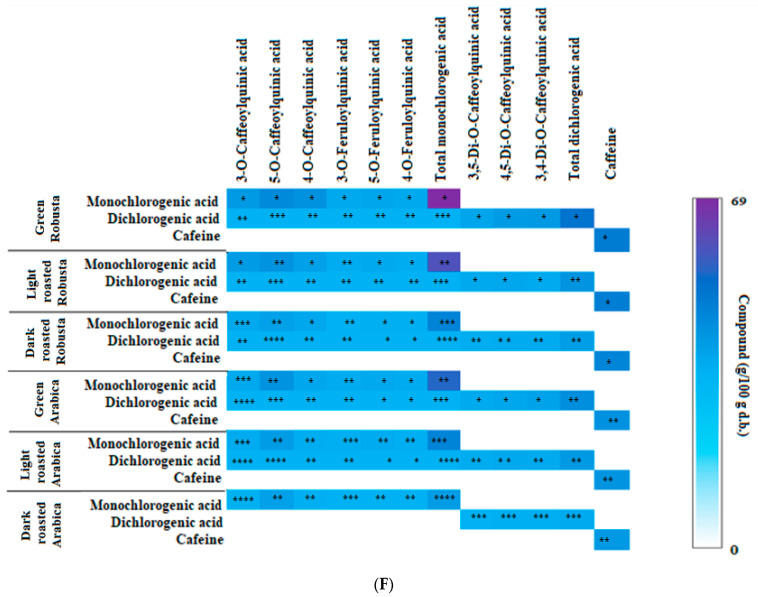
Concentration of mono-, dichlorogenic acids, and caffeine in fractions of coffee extracts after in vitro digestion: (**A**) in the stomach; (**B**) in the duodenum; (**C**) in the large intestine; (**D**) in the colon; (**E**) in the large intestine in the presence of probiotic bacteria; (**F**) in the colon in the presence of probiotic bacteria. The heatmap summarizes the concentration of compounds in isolated fractions. Each heatmap cell shows the concentration of compounds in isolated fractions as the intensity of a chromatic scale from white, as a value of zero, to dark violet, for high values. The left-hand scatterplot displays the coffee type used for fraction preparation. The upper scatterplot displays the names of individual compounds in the fraction. *^,^ **^,^ ***^,^ **** An equal number of markers in cells within one column means no statistical difference between concentrations at *p* < 0.05.

Qualitative and quantitative analyses of the chlorogenic acids and caffeine in the fractions of coffee extracts available during in vitro digestion were performed. Figure 2A shows the profile of mono- and dichlorogenic acids, as well as caffeine, after in vitro digestion in the stomach. A simulation of the digestion of mono- and dichlorogenic acids and caffeine fractions showed that, during the 1.5 h of digestion, the concentration of CQAs decreased by 20% and that of feruloylquinic acids (FChs) decreased by 5% (both belonging to monochlorogenic acids), compared with their pre-digestion values [16].

The level of dichlorogenic acids decreased by 10%, while the caffeine content decreased by 20%. The results suggest that coffee fractions consumed in this form, compared with whole coffee extracts, are characterized by higher gastric availability, probably due to the lack of a larger amount of dietary fiber that bound to the assigned compounds at low pH in the simulated stomach, as we showed in a previous study [16]. Since chlorogenic acids and caffeine are largely absorbed in the stomach in vivo, this form of administration may ensure faster absorption and physiological effects.

After the passage of the coffee fractions from the stomach to the small intestine, the samples were digested for 3 h, and then, their composition was analyzed again. Figure 2B shows the profile of mono- and dichlorogenic acids, as well as caffeine, after in vitro digestion in the duodenum. At this phase, it was found that, after in vitro digestion at alkaline pH, weak hydrolysis of the ester and hydrogen bonds occurred, resulting in the release of additional amounts of chlorogenic acids and caffeine. The content of CQA isomers increased by 5–7% and that of FCh isomers increased by 4–10%, while that of diCQA increased by 3–5%, and this increase was greater when analyzing fractions from more intense roasting. This means that chlorogenic acids are, to a greater extent, trapped in the macromolecular fraction of coffee beans in dark-roasted coffees. For comparison, the concentration of caffeine increased by 5% for all degrees of roasting. After another 3 h, samples were taken again, and Figure 2C shows the profiles of mono- and dichlorogenic acids, and caffeine after in vitro digestion in the large intestine. In the case of monochlorogenic acids of both coffee species, the concentration increased by 7% in the green coffee fractions, by 14% in the light-roasted fractions, and by 10% in the dark-roasted fractions, and that of the FCh isomers increased by 5, 10, and 15%, respectively, compared with digestion in the stomach. The level of dichlorogenic acids increased by 8%, 15%, and 20%, respectively, and that of caffeine increased by 6–8%.

In vitro digestion in the “colon” was carried out for the next 9 h, and samples were taken after digestion in the colon phase. The results are shown in Figure 2D. In all roasted samples of mono- and dichlorogenic acids, an increase in the concentration of the tested compounds was observed, compared not only with the earlier sections of the digestive tract but also with the initial concentrations before digestion [16]. It can therefore be assumed that, at this phase of digestion, the chlorogenic acids become available again in the amounts they were before digestion and the bonds forming adducts with macromolecules formed in the stomach are broken down. In the case of the monochlorogenic acid fraction of light-roasted Robusta, the content of CQA isomers increased from 36.91 to 41.36 g/100 g d.b. and that of Arabica increased from 17.56 to 20.06 g/100 g d.b., while for dark-roasted coffees, the increase was from 5.21 to 26.57 g/100 g d.b. and from 8.91 to 12.88 g/100 g d.b., respectively [16]. The caffeine concentrations increased by 5–10% compared with that at the start of digestion.

The metabolism of selected strains of probiotic bacteria in the post-digestion section in the “small intestine” resulted in higher release of mono- and dichlorogenic acids in comparison with in vitro digestion without bacteria. With a longer digestion time in the “colon”, the release of mono- and dichlorogenic acids progressed. Figure 2E,F show the profile of mono- and dichlorogenic acids, and caffeine after in vitro digestion in the “large intestine” and “colon” with LAB of selected strains. In the case of caffeine, its concentrations were higher by about 12% after 3 h in the “large intestine” and by 6–10% after 9 h in the “colon”. The highest concentrations of chlorogenic acids in the mono- and dichlorogenic acid fractions were characterized by in vitro digestion of preparations from green Robusta, while the highest increase compared with the concentration before in vitro digestion was characterized by dark-roasted coffees [16]. In the case of monochlorogenic acids isolated from the green Robusta extract, the content of their isomers increased by about 18 g/100 g d.b. and that from Arabica increased by 10 g/100 g d.b., while for light-roasted coffees, it increased by 23 g/100 g d.b. for Robusta and by 10 g/100 g d.b. for Arabica. Even for dark-roasted coffee beans, there was an increase in their concentration to about 50 g/100 g d.b. The concentrations of free dichlorogenic acids in the fractions from green coffees digested in the presence of probiotics increased by about 14 g/100 g d.b. for Robusta and by 7–8 g/100 g d.b. for Arabica, and for diesters present in the fraction from light roasted coffees, they increased by about 8 and 9 g/100 g d.b. for Robusta and Arabica, respectively, while for the dark-roasted coffees, they increased by out 5 I 3 g/100 g d.b. for Robusta and Arabica. Previous work [14], as well as studies by Castaldo et al. [28], clearly showed that, during digestion in the large intestine and colon, especially with simulated activity of the intestinal microflora, CQAs were released in amounts of 10–15%, which were previously incorporated into melanoidins during roasting of coffee beans. This may have a topical protective effect and may provide important health benefits when absorbed by entero- and colonocytes, including the prevention of many diseases such as colorectal cancer, obesity, cardiovascular disease, and diabetes.

### 3.3. Interactions and Affinity of BChE and Fractionated Coffee Extracts Digested In Vitro

**Figure 3:**
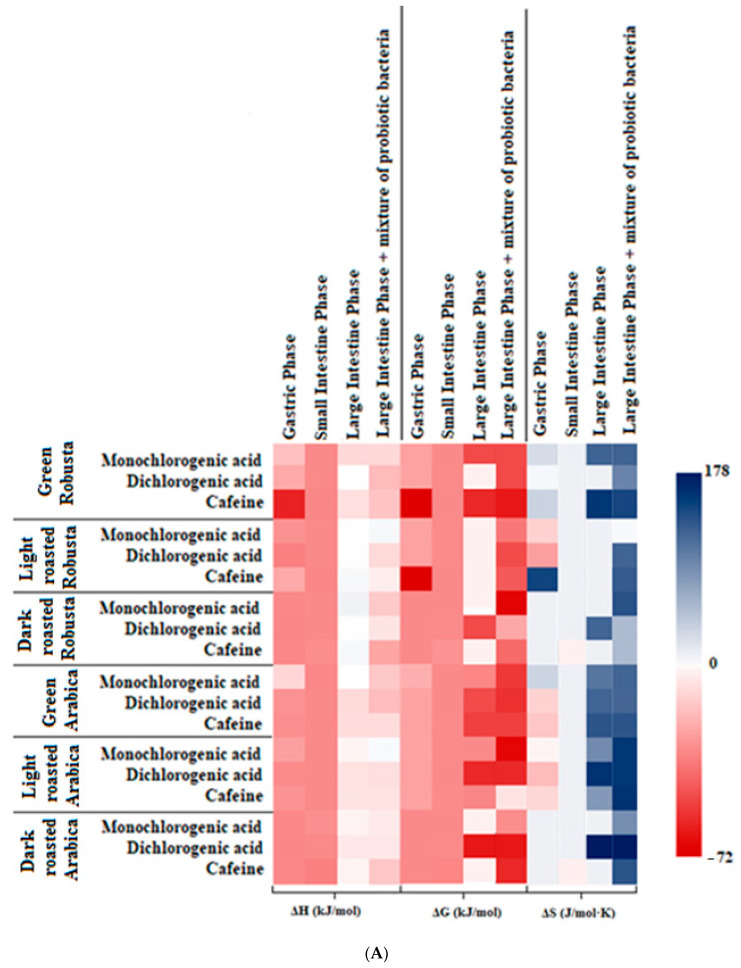

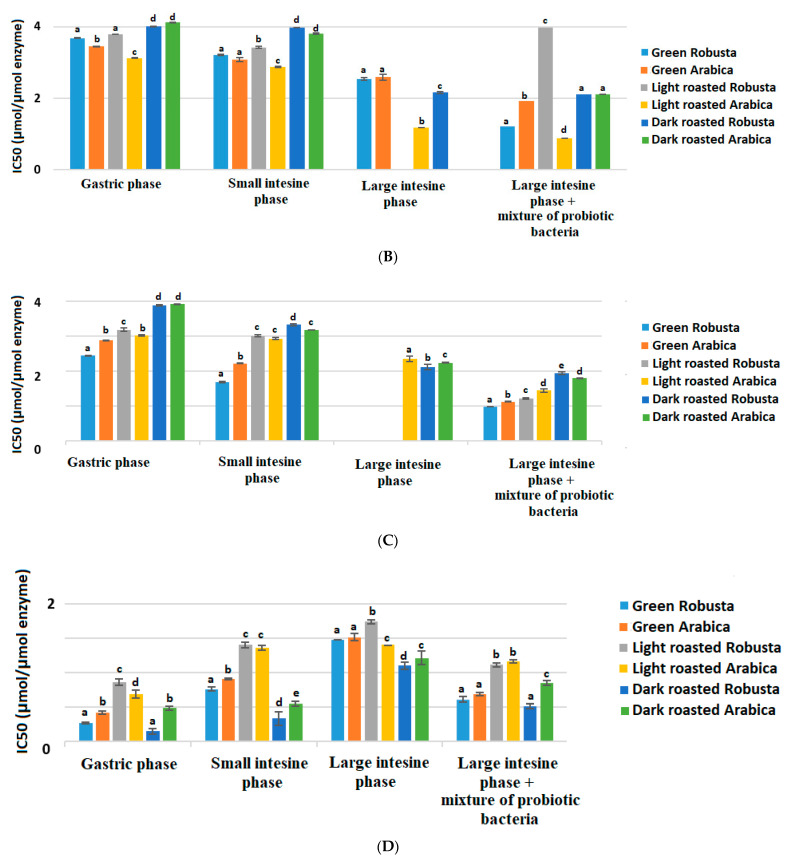
(**A**) Thermodynamic parameters of interactions of fractions isolated from coffee extracts digested in vitro with BChE (EC 3.1.1.8) and IC50 values for fractions of coffee extracts digested in vitro causing a reduction in BChE (EC 3.1.1.8) activity by 50%: (**B**) monochlorogenic acid fractions; (**C**) dichlorogenic acid fractions; (**D**) caffeine fractions. ^a–e^ Values are expressed as an average value ± SD; *n* = 6; different letters a–e in one section correspond to significant differences (*p* < 0.05).

The results of the analysis of the interaction of BChE and the fractions of coffee extracts—mono- and dichlorogenic acids, and caffeine—sampled before and after in vitro digestion are given in Table 1 and Figure 3, respectively.

The binding constant (K_A_) showed a large variation depending on the coffee fraction in the range of 1.92–58.41 × 103 L/mol for the fraction of monochlorogenic acids from light-roasted Arabica and dichlorogenic acids from dark-roasted Arabica. The lowest K_A_ were found for the dark-roasted Robusta coffee fractions. In competitive inhibition, an inhibitor binds to the enzyme in a manner that prevents the substrate binding. The inhibition constant Ki of nondigested fractions isolated from coffee extracts indicated that these fractions bound to BChE (EC 3.1.1.8) as competitive inhibitors and had aligned and low Ki values in the range of 0.13–0.88 μmol/L for the fractions of monochlorogenic acids isolated from dark-roasted Arabica and green Robusta (Table 1).

All fractions, apart from the caffeine fraction, isolated from the green Robusta extract showed an exothermic character in the interactions with BChE and a negative enthalpy change (ΔH). The change in entropy (ΔS) was positive in all cases, except for caffeine isolated from green Robusta, which may indicate high rotation of functional groups and conformational changes in the cyclic groups during docking, resulting from spatial adjustment to the structure of the enzyme.

**Figure 4:**
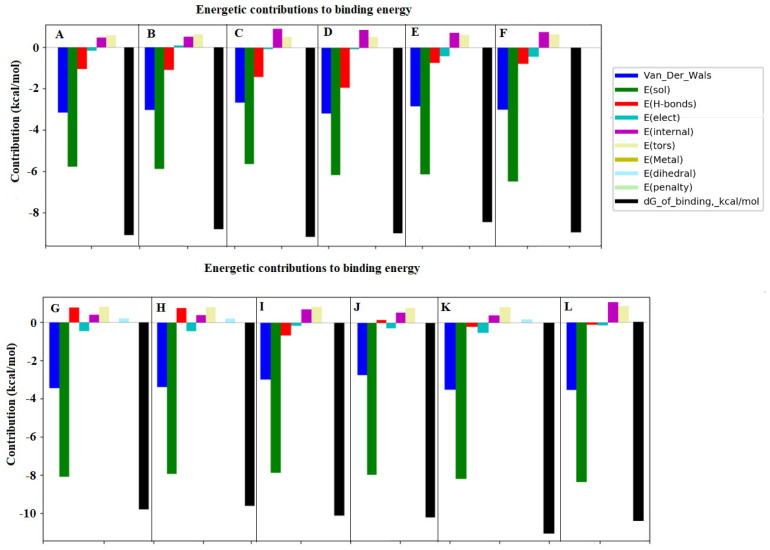
The results of docking simulation and the energy values of the interactions that make up the total binding energy of the ligand with BChE. (**A**) 5-CQA, 4-CQA, and 3-CQA; (**B**) 5-CQA, 3-CQA, and 4-CQA; (**C**) 4-CQA, 5-CQA, and 3-CQA; (**D**) 4-CQA, 3-CQA, and 5-CQA; (**E**) 3-CQA, 5-CQA, and 4-CQA; (**F**) 3-CQA, 4-CQA, and 5-CQA; (**G**) 3,4-DiCQA, 4,5-DiCQA, and 3,5-DiCQA; (**H**) 3,4-DiCQA, 3,5-DiCQA, and 4,5-DiCQA; (**I**) 3,5-DiCQA, 3,4-DiCQA, and 4,5-DiCQA; (**J**) 3,5-DiCQA, 4,5-DiCQA, and 3,4-DiCQA; (**K**) 4,5-DiCQA, 3,4-DiCQA, and 3,5-DiCQA; (**L**) 4,5-DiCQA, 3,5-DiCQA, and 3,4-DiCQA.

The affinity range (ΔG) was from −30.23 to −15.28 kJ/mol for the dichlorogenic acid and caffeine fractions of green Arabica extract, respectively (Table 1). Dichlorogenic acids exhibited high affinity for the enzyme, which is consistent with the docking simulation results, where high affinity (total energetic contribution) of diesters was demonstrated (Figure 4I–K). The affinity for the enzyme increased during digestion and was on average the highest after large intestine digestion, supported by the presence of probiotics (Figure 3A).

Our study has shown that, before digestion, the highest inhibition activity was observed for the dichlorogenic acid fraction, while the lowest was observed for the monochlorogenic acid fraction (Table 1). Importantly, all fractions showed an inhibition of more than 50%, which indicates high activity towards BChE side binding at physiological concentrations.

After digestion, the reaction enthalpy (ΔH) and ΔG were reduced compared with the pre-digestion values (Table 1, Figure 3A). The ΔH after digestion in the stomach resulted in amounts from −60.29 to −13.06 kJ/mol for the caffeine fraction isolated from green Robusta and for the monochlorogenic acids isolated from green Arabica. As digestion progressed, the enthalpy of the reaction between the enzyme, and the nutraceutics was at a similar level, amounts from −36 to −32 kJ/mol, but after digestion in the large intestine, it increased by about 70–80%. The affinity of the substrates and the entropy of interactions (ΔS) showed similar tendency. Interestingly, during digestion, all fractions showed affinity for BChE up to the stage of the large intestine, where the lack of affinity for BChE was exhibited by some fractions, mostly originating from Robusta, but the fractions digested in the presence of probiotics were characterized by high affinity, more beneficial in the case of isolates from Arabica (Figure 3A).

Our study showed that the IC50 value of digested coffee extracts was statistically lower for caffeine fractions compared with other fractions isolated from coffee extracts (Figure 3B–D). The fractions of monochlorogenic acids showed higher IC50 after digestion in the stomach and small intestine, amounting to about 3–4 μmol/μmol of the enzyme (Figure 3B), and was on average lower after digestion in the large intestine in the presence of lactic acid bacterial strains.

It was observed that dichlorogenic acids isolated from coffee extracts bound to BChE after digestion in the stomach and small intestine and in the large intestine in the presence of lactic acid bacteria but were digested without bacteria from extracts of light-roasted Arabica and the dark-roasted version of both species of coffee. In Arabica, chlorogenic acids were degraded more rapidly than in Robusta, and the compounds responsible for the interactions of BChE with the diester fraction from light-roasted Arabica may include the early products of chlorogenic acid degradation, as well as the products of the Maillard reactions [29,30].

After digestion in the large intestine in the presence of probiotic bacteria, the concentration needed to reduce the BChE activity by 50% was halved compared with that in the small intestine sample and was in the range of 0.98–1.95 μmol/μmol of the enzyme in the case of the green and dark-roasted Robusta diester fractions (Figure 3C).

Our study has shown that caffeine fractions isolated from coffee extracts, unlike dichlorogenic acid fractions, bound to BChE after all stages of in vitro digestion (Figure 3A,D). In addition, the lowest concentration of caffeine fractions causing BChE inhibition by 50% was observed compared with other digested fractions (Figure 3B–D). The caffeine fractions showed the highest BChE inhibition activity after digestion in the stomach, with IC50 values in the range of 0.12–0.69 μmol/μmol of the enzyme, in the case of the fraction isolated from dark- and light-roasted Robusta (Figure 3D). During further digestion in the small intestine, the activity decreased and the concentration of IC50 increased to the range of 0.27–1.12 μmol/μmol of the enzyme, for the fractions isolated from dark- and light-roasted Robusta. During digestion in the large intestine, the IC50 levels increased, while the presence of probiotic bacteria increased the ability of the caffeine fraction to inhibit enzyme activity. Farah et al. [31] found that, after the administration of green coffee supplements, serum concentrations of individual hydroxycinnamic acids were 5–10 μmol/L, and this level was maintained for up to 8 h, which is the effect of gradual absorption in different sections of the gastrointestinal tract. In our study, the concentration of BChE was 2 × 10^−6^ mol/L, i.e., about 1700 U/L, thus at the level of physiological concentration [32]. The nutraceutics’ concentrations in the measuring cell of an ITC apparatus after dilution at the time of injection were approximately 2 μmol/L, similar that of to the enzyme. Thus, for the most active analyzed BChE inhibitors derived from coffee beans, IC50 was in the range of physiologically occurring concentrations.

### 3.4. Simulation of Docking BChE and In Vitro Digested Coffee Fractions

In previous work, the molecular modeling of interactions of individual coffee nutraceutics with BChE was conducted. Adequate for the purposes of this study, in which the properties of the enzyme activity inhibition by fractions of coffee extracts were analyzed, docking was performed with two mixtures of three isomers of CQAs or diCQAs, where the docking sequence of the isomers was determined and a different docking sequence was used within each of the two families of isomers. This approach was used to investigate whether the isomers are competitive with each other when docking to the enzyme at the catalytic site. The binding of BChE with monochlorogenic acids was taken into account in six different orders—3-CQA, 4-CQA, and 5-CQA; 3-CQA, 5-CQA, and 4-CQA; 4-CQA, 3CQA, and 5CQA; 4-CQA, 5-CQA, and 3-CQA; 5-CQA, 3-CQA, and 4-CQA; and 5-CQA, 4-CQA, and 3-CQA—and with dichlorogenic acids in five configurations—3,4-DiCQA, 3,5-DiCQA, and 4,5-DiCQA; 3,4-DiCQA, 4,5-DiCQA, and 3,5-DiCQA; 3,5-DiCQA, 3,4-DiCQA, and 4,5-DiCQA; 3,5-DiCQA, 4,5-DiCQA, and 3,4-DiCQA; and 4,5-DiCQA, 3,4-DiCQA, and 3,5-DiCQA. The amino acids found in the catalytic triad of BChE, namely SER198, HIS438, and GLU325, were considered the docking site. Furthermore, the BChE hydrolytic activity depends on their availability in binding with the amino acid residues forming the pocket with ACh, in which the peripheral anionic site occurs, which involves the ASP70 and TYR332 side chains, followed by the choline binding site with TRP82 and the oxonic site containing GLY116, GLY117, and ALA199. The active site forms a complex with the neurotransmitter and forces ACh to rotate. After rotation and binding to the acyl pocket containing LEU286 and VAL288, hydrolysis of ACh to choline and the acyl group is possible via a catalytic triad [33,34].

**Figure 5:**
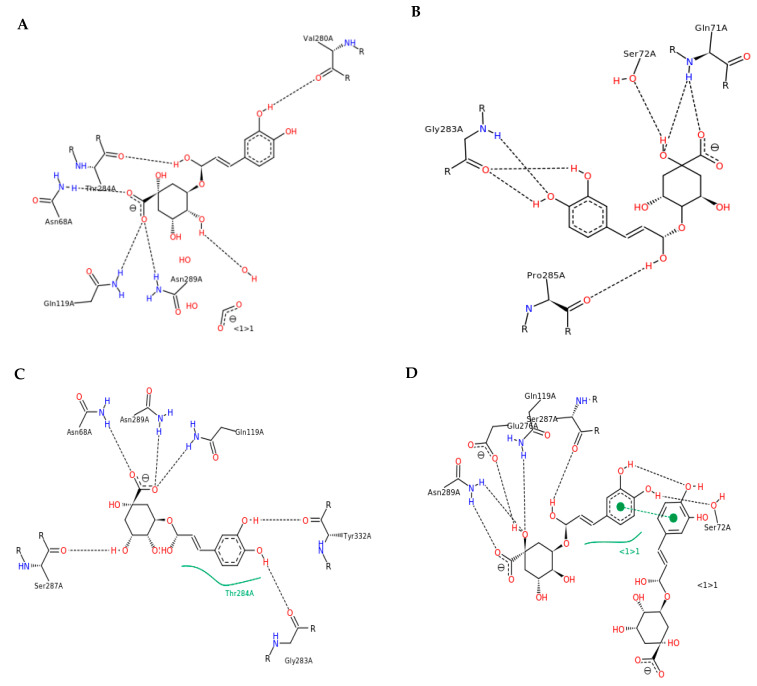

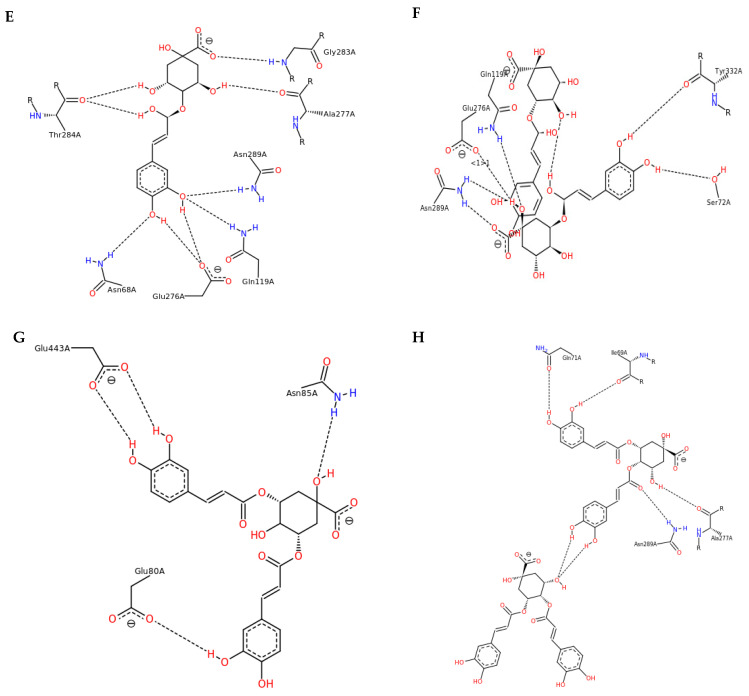

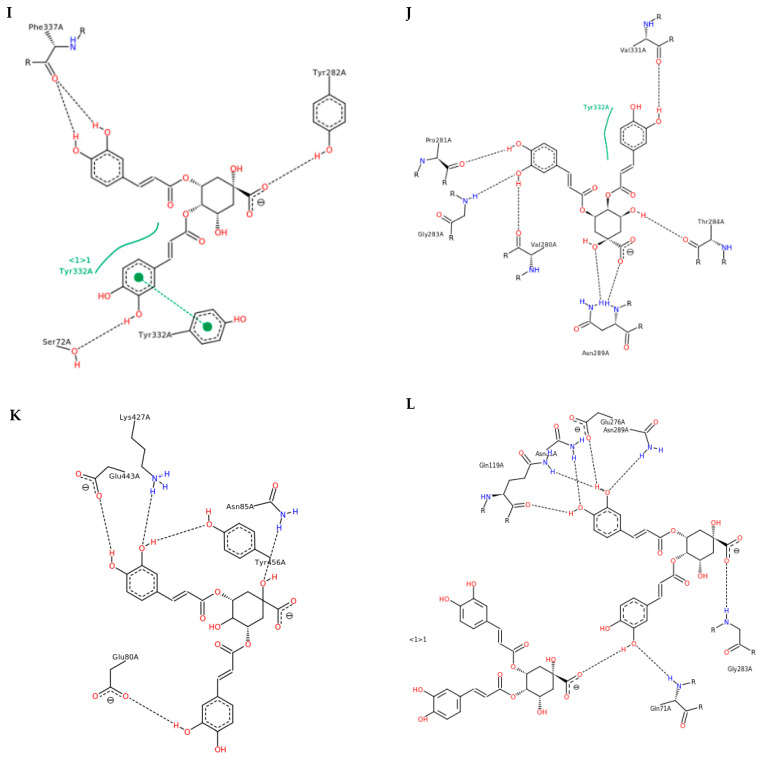
The results of docking simulation and 2D model of the ligand interaction with BChE. (**A**) 5-CQA, 4-CQA, and 3-CQA; (**B**) 5-CQA, 3-CQA, and 4-CQA; (**C**) 4-CQA, 5-CQA, and 3-CQA; (**D**) 4-CQA, 3-CQA, and 5-CQA; (**E**) 3-CQA, 5-CQA, and 4-CQA; (**F**) 3-CQA, 4-CQA, and 5-CQA; (**G**) 3,4-DiCQA, 4,5-DiCQA, and 3,5-DiCQA; (**H**) 3,4-DiCQA, 3,5-DiCQA, and 4,5-DiCQA; (**I**) 3,5-DiCQA, 3,4-DiCQA, and 4,5-DiCQA; (**J**) 3,5-DiCQA, 4,5-DiCQA, and 3,4-DiCQA; (**K**) 4,5-DiCQA, 3,4-DiCQA, and 3,5-DiCQA; (**L**) 4,5-DiCQA, 3,5-DiCQA, and 3,4-DiCQA.

The results of the docking simulations conducted with the mixture of compounds are presented in Figure 4A–L and Figure 5A–L, showing, for the projection clarity, only the complexes with the compound that exhibited the strongest interactions within a given approach.

The graphs of the energetic contribution to the binding energy represent the energies of different kinds of interactions coming from the docking of all three isomers. The docking simulation showed that BChE interacting with monochlorogenic acids in the order of 5-CQA, 4-CQA, and 3-CQA bound with 5-CQA mainly through the hydrogen bonds in Val280, ASN68, ASN289, GLN119, and THR284 (Figure 4A and Figure 5A), while in the case with the order of 5-CQA, 3-CQA, and 4-CQA, the docking of 4-CQA involved GLN71, GLY283, PRO285, and SER72 (Figure 4B and Figure 5B). Considering the docking order of 3-CQA, 5-CQA, and 4-CQA, the interactions with 4-CQA were mainly related to GLY283, ALA277, ASN289, ASN68, GLN119, GLU276, and THR284 (Figure 4E and Figure 5E), and for that with the order of 3-CQA, 4-CQA, and 5-CQA, the complex with two molecules of 5-CQA was formed with ASN289, GLN119, GLU276, SER72, and TYR332 (Figure 4F and Figure 5F). The other two sequences (4-CQA, 3-CQA, and 5-CQA, and 4-CQA, 5-CQA, and 3-CQA; Figure 4C,D) show higher binding energies (Table 2).

It was noticed that the mixture of three monochlorogenic acids increased the binding energy by about 1–6 kJ/mol compared with previous studies of individual CQAs [17]. This is related to the occurrence of not only the hydrogen bonds but also the hydrophobic interactions associated with SER72 (Figure 4D) and THR284 (Figure 4C). In addition, during docking with a sequence of 4-CQA, 5-CQA, and 3-CQA, π-π interactions with SER72 were also observed (Figure 4D). This suggests additional interactions that promote stabilization of the complex and enable binding to the active side at TYR322 (Figure 4C), which is located in the anionic pocket and may prevent ACh from reaching the active site of the enzyme. The sequence of monochlorogenic acids 4-CQA, 3-CQA, and 5-CQA also bound with SER287 in the catalytic triad at the site of the acyl group [35,36]. Hydrogen interactions with GLY283 in the pocket of oxyanion were also involved (Figure 4C) [35,37].

Furthermore, in the case of dichlorogenic acids, the compounds showed a higher affinity for the enzyme than individual diCQAs, at the level of −47.04–−43.23 kJ/mol, except for 3,5-diCQA, in which the ΔG was −49.39 kJ/mol [17]. However, diesters as a mixture had higher affinity for the enzyme than CQAs (Table 2, Figure 4G–K). The highest affinity was characterized by dichlorogenic acids docking with the order of 4,5-DiCQA, 3,4-DiCQA, and 3,5-DiCQA (Table 2). It can be seen that the complex is characterized by hydrogen bonds with LYS427, GLU443, ASN85, TYR456, and GLU80 (Figure 4K).

It can be observed that the relationship is valid for almost all dockings of the mixtures: the first compound docking has the strongest possibility of binding to the enzyme, while subsequent dockings have lower binding energies. When using different docking orders, the same compounds show altered binding energies. This proves the dependence of the binding energy on the surrounding compounds and the competitive nature of the analyzed ligands. It is worth emphasizing that higher binding energies (affinity) were observed for the docking of the mixtures compared with the docking of single chlorogenic acids or even the coffee extracts analyzed in previous work [17]. This indicates that a fraction of several active compounds with high concentrations is a more effective enzyme inhibitor than either a single compound or an extract in which the active compounds remain highly diluted.

## 4. Conclusions

Studies of the interactions of fractionated coffee extracts with BChE showed the formation of stable complexes via the main ingredients of the fractions at the active sites of the enzyme, as well as showed inhibition of the activity of the enzyme for acetylcholine hydrolysis. In vitro digestion of fractions isolated from coffee extracts, containing mainly mono- and dichlorogenic acids or caffeine, accompanied by other coffee compounds, increased the affinity for enzymes of digested preparations after the large intestine phase, in particular in the presence of the selected strains of lactic acid bacteria. This suggests that the fractions isolated from coffee extracts after digestion can effectively inhibit BChE activity. The obtained results indicate the potential health-promoting effects, resulting from enzyme inhibition, of coffee extracts, especially their concentrated fractions in the human body. The highest potential has been shown using isolates of dichlorogenic acids from green Arabica. This method of preparation could be used for the development of functional foods proposed for neurodegenerative and metabolic disorders prophylaxis. In subsequent studies, tests on human cells are planned to confirm the supposition.

## Figures and Tables

**Table 1:** Parameters of interactions of fractionated coffee extracts with BChE (EC 3.1.1.8) and their activity as BChE (EC 3.1.1.8) inhibitors.

Fraction from Coffee Extract	K_A_ × 10^3^ (L/mol)	∆H (kJ/mol)	∆G (kJ/mol)	∆S (J/mol × K)	Inhibitory Activity (%)	μmol/μmol Enzyme IC50	K*_i_* (μmol/L) K*_M_* ACh 35.9
Green Arabica							
Monochlorogenic acid	14.06 ± 0.95 ^a^	−0.70 ± 0.03 ^a^	−18.67 ± 0.03 ^a^	62.65 ± 0.01 ^a^	51.77 ± 0.03 ^a^	1.25 ± 0.01 ^b^	0.24 ± 0.02 ^a^
Dichlorogenic acid	12.35 ± 2.35 ^a^	−0.75± 0.02 ^a^	−30.23 ± 0.02 ^b^	95.36 ± 0.02 ^b^	86.37 ± 0.10 ^b^	0.86 ± 0.00 ^a^	0.32 ± 0.01 ^a^
Caffeine	40.58 ± 6.98 ^b^	−0.31 ± 0.01 ^b^	−15.28 ± 0.02 ^a^	48.42 ± 0.02 ^c^	53.20 ± 0.02 ^a^	1.20 ± 0.01 ^b^	0.56 ± 0.03 ^b^
Light roasted Arabica							
Monochlorogenic acid	1.92 ± 0.20 ^a^	−1.17 ± 0.09 ^c^	−25.41 ± 0.03 ^c^	78.41 ± 0.05 ^d^	54.83 ± 0.02 ^a^	1.59 ± 0.01 ^c^	0.34 ± 0.01 ^a^
Dichlorogenic acid	47.15 ± 1.35 ^b^	−0.54 ± 0.03 ^a^	−21.81 ± 0.02 ^c^	60.80 ± 0.05 ^a^	74.99 ± 0.06 ^b^	1.35 ± 0.01 ^b^	0.62 ± 0.02 ^b^
Caffeine	41.48 ± 2.25 ^b^	−0.11 ± 0.02 ^d^	−20.48 ± 0.02 ^c^	65.89 ± 0.02 ^a^	73.03 ± 0.04 ^b^	1.51 ± 0.01 ^c^	0.57 ± 0.02 ^b^
Dark roasted Arabica							
Monochlorogenic acid	27.10 ± 1.10 ^a^	−0.39 ± 0.02 ^b^	−22.39 ± 0.04 ^c^	71.16 ± 0.02 ^d^	71.44 ± 0.10 ^b^	1.91 ± 0.01 ^d^	0.13 ± 0.04 ^c^
Dichlorogenic acid	58.41 ± 3.25 ^b^	−0.05 ± 0.04 ^d^	−20.05 ± 0.02 ^c^	64.69 ± 0.05 ^a^	95.40 ± 0.12 ^c^	1.48 ± 0.00 ^b^	0.25 ± 0.01 ^a^
Caffeine	23.90 ± 1.45 ^a^	−0.31 ± 0.01 ^b^	−21.31 ± 0.01 ^c^	67.96 ± 0.04 ^a^	75.56 ± 0.06 ^b^	2.29 ± 0.01 ^d^	0.67 ± 0.03 ^b^
Green Robusta							
Monochlorogenic acid	1.95 ± 0.20 ^a^	−1.22 ± 0.01 ^c^	−25.41 ± 0.12 ^c^	78.24 ± 0.03 ^d^	66.32 ± 0.03 ^a^	1.29 ± 0.00 ^b^	0.88 ± 0.01 ^d^
Dichlorogenic acid	53.05 ± 2.25 ^b^	−1.10 ± 0.02 ^c^	−22.15 ± 0.02 ^c^	68.09 ± 0.06 ^a^	75.40 ± 0.06 ^b^	0.70 ± 0.00 ^a^	0.72 ± 0.00 ^d^
Caffeine	55.56 ± 2.55 ^b^	5.44 ± 0.03 ^e^	−21.01 ± 0.03 ^c^	−50.36 ± 0.02 ^e^	64.85 ± 0.01 ^a^	1.30 ± 0.01 ^b^	0.32 ± 0.02 ^a^
Light roasted Robusta							
Monochlorogenic acid	2.51 ± 0.15 ^a^	−8.88 ± 1.15 ^f^	−17.48 ± 0.02 ^a^	27.82 ± 0.06 ^f^	62.75 ± 0.02 ^a^	1.56 ± 0.00 ^c^	0.38 ± 0.03 ^a^
Dichlorogenic acid	10.26 ± 0.35 ^a^	−16.54 ± 3.55 ^g^	−30.19 ± 0.10 ^b^	44.15 ± 0.09 ^b^	98.44 ± 0.10 ^c^	1.30 ± 0.01 ^b^	0.23 ± 0.02 ^a^
Caffeine	43.57 ± 2.20 ^b^	−0.62 ± 0.00 ^a^	−21.60 ± 0.03 ^c^	67.86 ± 0.05 ^a^	73.72 ± 0.03 ^b^	1.11 ± 0.01 ^b^	0.43 ± 0.03 ^c^
Dark roasted Robusta							
Monochlorogenic acid	2.24 ± 0.55 ^a^	−1.20 ± 0.09 ^c^	−27.98 ± 0.10 ^c^	86.62 ± 0.02 ^d^	80.77 ± 0.02 ^b^	2.21 ± 0.01 ^d^	0.53 ± 0.01 ^c^
Dichlorogenic acid	7.86 ± 1.15 ^a^	−8.90 ± 0.04 ^f^	−29.06 ± 0.12 ^c^	65.21 ± 0.03 ^a^	97.86 ± 0.10 ^c^	1.42 ± 0.01 ^b^	0.14 ± 0.08 ^a^
Caffeine	9.43 ± 2.35 ^a^	−0.20 ± 0.01 ^d^	−28.52 ± 0.11 ^c^	91.60 ± 0.05 ^b^	84.68 ± 0.02 ^b^	1.57 ± 0.01 ^c^	0.46 ± 0.04 ^b^

Values are expressed as an average value ± SD; *n* = 6; different letters ^a–g^ in one column correspond to significant differences (*p* < 0.05).

**Table 2:** Binding energy calculated from the docking simulation of mixtures of three chlorogenic acid isomers (compounds that dominated in monochlorogenic or dichlorogenic acid fractions obtained from coffee extracts). Steps correspond to docking of phenolics according to the given sequence.

Sequence	Energy (kJ/mol)			
	Step 1	Step 2	Step 3	Average Step
3-CQA, 4-CQA, 5-CQA	−41.51	−45.86	−33.47	−40.28
3-CQA, 5-CQA, 4-CQA	−41.51	−38.07	−35.15	−38.24
4-CQA, 3-CQA, 5-CQA	−42.34	−45.44	−36.02	−41.27
4-CQA, 5-CQA, 3-CQA	−42.34	−40.58	−38.99	−40.64
5-CQA, 3-CQA, 4-CQA	−38.66	−34.35	−35.02	−36.01
5-CQA, 4-CQA, 3-CQA	−38.66	−40.92	−37.99	−39.19
3,4-DCQA, 3,5-DCQA, 4,5-DCQA	−50.75	−42.47	−37.53	−43.58
3,4-DCQA, 4,5-DCQA, 3,5-DCQA	−50.75	−37.99	−40.96	−43.23
3,5-DCQA, 3,4-DCQA, 4,5-DCQA	−50.42	−41.17	−41.59	−44.39
3,5-DCQA, 4,5-DCQA, 3,4-DCQA	−50.42	−42.05	−41.76	−44.74
4,5-DCQA, 3,4-DCQA, 3,5-DCQA	−49.16	−45.56	−46.40	−47.04
4,5-DCQA, 3,5-DCQA, 3,4-DCQA	−49.12	−45.52	−41.04	−45.23

## Data Availability

Data available on request.
